# Exploring BCL2 regulation and upstream signaling transduction in venetoclax resistance in multiple myeloma: potential avenues for therapeutic intervention

**DOI:** 10.1038/s41408-025-01215-x

**Published:** 2025-02-05

**Authors:** Rodrigo Fonseca, Yuan Xiao Zhu, Laura A. Bruins, Joseph Ahmann, Cecilia de Bonolo Campos, Esteban Braggio, Xianfeng Chen, Mariano Arribas, Susie Darvish, Seth Welsh, Erin Meermeier, Kiran K. Mangalaparthi, Richard K. Kandasamy, Greg Ahmann, J. Erin Wiedmeier-Nutor, Akhilesh Pandey, Marta Chesi, P. Leif Bergsagel, Rafael Fonseca

**Affiliations:** 1 Division of Internal Medicine, Mayo Clinic, AZ USA; 2 Division of Hematology and Medical Oncology, Mayo Clinic, AZ USA; 3 Division of Biomedical Statistics and Informatics, Department of Health Science Research, Mayo Clinic, Rochester, MN USA; 4 Department of Laboratory Medicine and Pathology, Mayo Clinic, Rochester, MN USA; 5 Manipal Academy of Higher Education, Manipal, Karnataka India

**Keywords:** Cancer therapeutic resistance, Myeloma

## Abstract

Investigating venetoclax (VTX) resistance in multiple myeloma (MM) is crucial for the development of novel therapeutic strategies to tackle resistance. We conducted a multi-omic characterization of established VTX-resistant isogenic human myeloma cell lines (HMCL) and primary MM patient samples pre- and post-VTX treatment. Transcriptomic and proteomic analysis revealed that resistance was largely associated with BCL-2 family protein dysregulation, including upregulation of anti-apoptotic proteins such as MCL-1, BCL-XL, BCL-2, and downregulation of pro-apoptotic members. Notably, the re-introduction of BIM into resistant cells restored VTX sensitivity and synergized with MCL-1 inhibitors. Upstream signaling pathways, including growth factor receptor tyrosine kinase (RTK) and phosphoinositide-3-kinase (PI3K) were implicated in this dysregulation. Simultaneous inhibition of MCL-1, BCL-XL, and upstream PI3K, RTK (FGF, EGF, and IGF1) mediated signaling enhanced VTX sensitivity. Post-translational modifications of MCL-1, particularly its stabilization via acetylation and phosphorylation, were investigated, although their inhibition only marginally increased VTX sensitivity. Lastly, the inhibition of AURKA and mitochondrial respiration also improved VTX sensitivity in some resistant HMCLs. Our findings suggest that combining VTX with MCL-1 and BCL-XL inhibitors or PIK3/RTK inhibitors holds potential for overcoming resistance. The study illustrates the importance of understanding molecular determinants of resistance to develop tailored therapeutic strategies.

## Introduction

The evasion of apoptosis represents a fundamental hallmark of cancer [1, 2]. Among the pathways that regulate apoptosis, the intrinsic (mitochondrial) pathway is governed by a complex interplay between pro-apoptotic and anti-apoptotic members of the BCL-2 family. Anti-apoptotic proteins, such as BCL-2, MCL-1, and BCL-XL, thwart apoptosis by sequestering both BH3-only proteins (e.g., BIM and PUMA) and effector pro-apoptotic proteins (e.g., BAX and BAK) [3, 4]. BH3 mimetic drugs, which resemble BH3 domains, bind to anti-apoptotic proteins, displacing the BH3-only proteins and inducing apoptosis through BAX/BAK activation [5].

Venetoclax (VTX), a BH3 mimetic, antagonist of BCL-2, has been approved for the treatment of chronic lymphocytic leukemia (CLL), acute myeloid leukemia (AML), and is undergoing clinical trials for the treatment of multiple myeloma (MM) [6–8]. Trials have shown significant antitumor activity in the disease subsets that present lymphoid features, such as those harboring t(11;14) translocations [9]. While MM remains an incurable disease, advances in the anti-MM therapeutic arsenal are essential [10–14]. However, as described with other anti-MM drugs, acquired resistance to VTX represents a clinical challenge. Therefore, a thorough understanding of the underlying mechanisms of resistance and the development of strategies to prevent or overcome such resistance are imperative.

VTX sensitivity has been correlated with high *BCL2* and low *BCL2L1* and *MCL1* gene expression, commonly associated with a t(11;14) and lymphoid biology [15–17]. Acquired VTX resistance has been linked to the upregulation of *BCL2L1* and *MCL1* [18, 19]. Moreover, *BCL2* mutations have been implicated in VTX resistance in CLL and AML, but not in MM [20, 21]. Therefore, our study aims to elucidate the underlying mechanisms contributing to acquired resistance to VTX in MM and develop strategies to overcome and prevent resistance, paving the way for more effective and durable therapeutic approaches in the battle against MM.

## Methods

### Ethics approval and consent to participate

The study was conducted in accordance with relevant guidelines and regulations and was approved by the Institutional Review Board under protocol 919-04. All samples were collected and tested following approval under this protocol. Informed consent was obtained from all participants.

### Cells and reagents

Characteristics of human myeloma cell lines (HMCLs) used in this study are detailed in the supplementary material, along with antibodies and compounds used for experiments (Table S1). Table S1 summarizes molecular characteristics including canonical translocation and TC classification, along with the tissue source of origin. Cell lines were authenticated by CNV analysis, as described previously [22]. Cells were maintained in RPMI-1640 media, supplemented with 5% fetal calf serum and antibiotics. Primary human MM cells were recovered from bone marrow aspirates collected from Mayo Clinic sites. After collection, CD138+ cells were isolated by immunomagnetic bead selection (RoboSep; Stemcell Technologies, Vancouver, BC, Canada). Informed consent was obtained under Institutional Review Board approval (IRBs 919-04, 15-009436, 18-003198, 2207-02) in accordance with the Declaration of Helsinki.

### Establishment of VTX-resistant HMCLs

Four VTX-resistant HMCLs were generated by subjecting VTX-sensitive cell lines, KMS12PE, OCIMY7, SKMM2, and OCIMY5, to prolonged VTX exposure. The initial concentration of VTX was set at a half-maximal inhibitory concentration (IC50) dose specific to each cell line. Cell lines were maintained at each concentration until they exhibited normal growth rates, after which the VTX concentration was increased. Identities of all resistant HMCLs generated were validated by fingerprint. Resistance was confirmed via cell viability assays evaluating IC50 values.

### Time-series experiments for protein modulation and half-life assessment

To assess MCL-1 modulation, cells were exposed to selected compounds (A-485, GNE-781, WP-1130, Okadaic acid, OA) for set durations. Post-treatment, cells were harvested, washed, and stored at −80 C. For protein half-life determination, cells were incubated in replicates at each time point of interest with 25 µg/ml of cycloheximide. For assessing half-life after treatment, cells were first pre-treated with the compound of interest for a predetermined duration and were then subjected to cycloheximide. The compound of interest remained present during cycloheximide exposure. Experiments were performed in duplicates.

### Immunoblotting and co-immunoprecipitation assay

Western blots were performed according to the manufacturer’s protocol. Briefly, 30 μg of protein was subjected to SDS-PAGE. Separated proteins were transferred onto PVDF membranes, blocked, and probed with primary antibodies overnight. Subsequently, membranes were incubated with horseradish peroxidase-conjugated secondary antibodies for signal detection. Protein bands were visualized using the Enhanced chemiluminescence (ECL) method. Co-immunoprecipitation was performed according to the manufacturer’s protocol using a Co-IP kit from Takara (San Jose, CA, USA).

### Cell viability assays

Cell viability and growth were assessed using the 3-(4,5-dimethylthiazol)-2,5-diphenyltetrazolium bromide (MTT) dye absorbance assay (Boehringer Mannheim) or CellTiter-Glo viability assay (Promega, Madison, WI, USA). Each experimental condition was performed in triplicates or quadruplicates and was repeated at least twice.

### DNA and RNA extraction

Total RNA and genomic DNA from HMCLs and CD138+ bone marrow cells from MM patients were isolated using RNeasy or the AllPrep DNA/RNA Kit (Qiagen, Geramantown, MD, USA).

### *mRNA* sequencing (mRNA-seq) and data analysis

mRNA sequencing was conducted using a capture-based approach, generating ~20 million reads per sample. The differential expression analysis used read counts derived from gene expression levels and included: (1) read count normalization; (2) model-dependent *p* values estimation; and (3) estimation of false discovery rate (FDR). The Kyoto Encyclopedia of Genes and Genomes (KEGG) was used for enrichment analysis of the differentially expressed genes. Additionally, mRNA-seq data underwent processing using the Mayo Clinic RNA-S pipeline, which aligns reads to the human hg19 genome build using TophAT V2.0.12, and quantified gene count via featureCounts v.1.4.4. EdgeR v2.6.2 identified differently expressed genes, with an FDR ≤ 0.05. Ingenuity pathway analysis (IPA, http://www.ingenuity.com/) assessed enriched pathways.

### Whole exome sequencing (WES)

WES was performed by Novogene, and data was analyzed by the bioinformatics core at Mayo Clinic. Pair-ended reads were subjected to Fastp for adapter-trimming and low base quality removal, then Aligned to human genome reference (HG38)(https://bio-bwa.sourceforge.net/bwa.shtml). Duplicated reads marked and base recalibrated by GATK (https://gatk.broadinstitute.org/hc/en-us), somatic mutations were called from paired samples by mutect2 (https://gatk.broadinstitute.org/hc/en-us/articles/360037593851-Mutect2) and then filtered by mutet2 module FilterMutectCalls. Mutations were anoted by SnpEff (http://pcingola.github.io/SnpEff/), VCF files were converted to MAF format by vcf2maf (https://github.com/mskcc/vcf2maf) and viewed by maftools.

### Quantitative proteomics analysis

Quantitative proteomic analysis of VTX-sensitive and resistant cell lines was performed as described previously [23]. Briefly, protein extraction was performed in 9 M urea and 50 mM triethyl ammonium bicarbonate lysis buffer supplemented with a protease inhibitor cocktail. Following reduction and alkylation, proteins were digested with Trypsin/Lys-C enzyme mix. Peptides were then labeled with TMTpro reagents and subsequently pooled and fractionated into 24 fractions using basic pH reversed-phase fractionation on an UltiMate 3000 HPLC system (Thermo Fisher Scientific, Waltham, MA, USA).

LC-MS/MS analysis was performed using an Orbitrap Eclipse Tribrid mass spectrometer connected to a Vanquish Neo liquid chromatography system (Thermo Scientific, San Jose, CA, USA). Online separation of peptides was performed using a trap and elute configuration. The mass spectrometer was operated in a data-dependent mode with 2.5 seconds cycle time. In the MS scan, the precursor ions were measured in the Orbitrap mass analyzer. Top abundant precursor ions were isolated by quadrupole for MS/MS analysis using an isolation width of 0.7 Da. High-energy collision-induced dissociation (HCD) was used for peptide backbone fragmentation at 34% normalized collision energy. MS/MS spectra were recorded in the Orbitrap at 30,000 resolution. Protein identification and quantitation were performed using Proteome Discoverer software version 3.0 (Thermo Scientific, San Jose, CA). Database searching was performed by Sequest HT search engine using human UniProt protein database with full tryptic cleavage specificity, two missed cleavages, precursor mass tolerance of 10 ppm, and fragment ion mass tolerance of 0.02 Da. Oxidation (methionine) and protein N-terminal acetylation were set as dynamic modifications, and TMTpro at peptide N-terminus, lysine, and carbamidomethylation (cysteine) were specified as fixed modifications. The false discovery rate was maintained at 1% at the protein level and peptide level using Percolator.

### Statistical analysis

We utilized several approaches and software tools to analyze the data. For cell viability assays, data were normalized in relation to untreated controls, and IC50 was calculated using non-linear regression analysis (GraphPad Prism, Version 9.5.1). Protein half-life was determined using the one-phase exponential decay non-linear regression analysis. SynergyFinder software was used for drug synergy analysis [24]. Student’s *t* test and ANOVA analysis were used to determine statistical differences between experimental conditions. Western Blot band density was calculated using iBright Analysis Software. Total protein content was standardized to housekeeping gene or using total protein normalization protocol. The Pearson correlation coefficient was calculated to examine the linear relationship between two continuous variables. Non-linear regression analysis was used to investigate the relationship between protein expression and RNAseq data using BlueSky Statistics. All statistical tests were chosen based on data meeting their assumptions, including normality and homogeneity of variance, which were confirmed prior to analysis. Variation is reported as the standard deviation or standard error of the mean.

## Results

### MM cells with acquired resistance to VTX demonstrated expression changes in BCL2 family members but no *BCL2* mutation

**Fig. 1:**
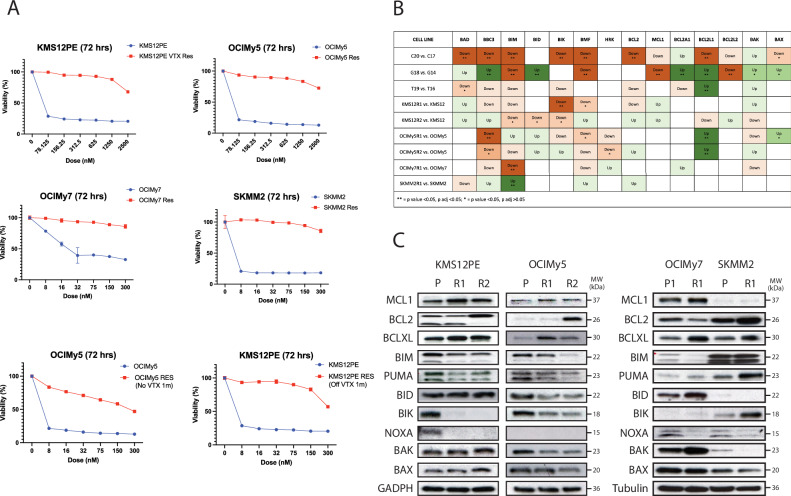
Assessment of venetoclax resistance and associated transcriptional and protein changes. **A** MTT assay results after 72-hour incubation, with viability normalized to untreated control. The viability of parental and venetoclax-resistant MM cell lines is displayed in the top and middle panels. Persistence of resistance after 1 month of venetoclax withdrawal is demonstrated in the bottom panel. **B** Table summarizing transcriptional changes (from mRNA-seq data) between isogenic and resistance-acquired cell lines, and three patient samples (C, G, and T) pre- and post-VTX exposure; darker colors indicate greater statistical significance. **C** Western Blot analysis of protein expression in parental and venetoclax-resistant cell lines.

Four HMCLs with acquired resistance to VTX were established by culturing corresponding VTX-responsive HMCLs in the presence of VTX (Fig. 1A upper and middle panels). Resistance persisted in two cell lines after withdrawing VTX for one and 3 months (Fig. 1A bottom panel; Supplementary Fig. S1). To explore underlying mechanisms, we performed transcriptomics analysis of all four isogenic sensitive/resistant HMCLs pairs and three MM patient samples collected before VTX treatment and after relapse or progression (Supplementary Data S1).

Since VTX is a BCL-2 antagonist, we first examined transcriptional changes in BCL2 family members. The most common changes included *BCL2L1* (BCL-XL) upregulation and downregulation of *BCL2L11* (BIM), *BBC3 (*PUMA), *BIK,* and *BMF* (Fig. 1B). The *BCL2*/*BCLXL* ratio was downregulated across 2/4 resistant cell lines and 2/3 relapsed patient samples (Supplementary Fig. S2). Western blot analysis (Fig. 1C) revealed that most resistant (R) HMCLs exhibited upregulated anti-apoptotic proteins (MCL-1 and BCL-XL) and downregulated BH3-only members including BIM, PUMA, BID, BIK, and NOXA compared with their corresponding parental (P) sensitive HMCLs. Interestingly, while *MCL-1* upregulation was not significant at the transcriptional level, upregulation was evident at the protein level. Downregulation of effector pro-apoptotic proteins (BAX and BAK) was also detected. BCL-2 expression was downregulated in two resistant cell lines collected after only three months of VTX exposure (KMS12PERes R1 and OCIMy5Res R1). After longer exposure, BCL-2 upregulation was seen in three R-HMCLs (KMS12PERes R2, OCIMy5Res R2, and SKMM2 R1). Downregulation of BIM also became more prominent in the resistant cell line harvested after longer VTX exposure.

**Fig. 2:**
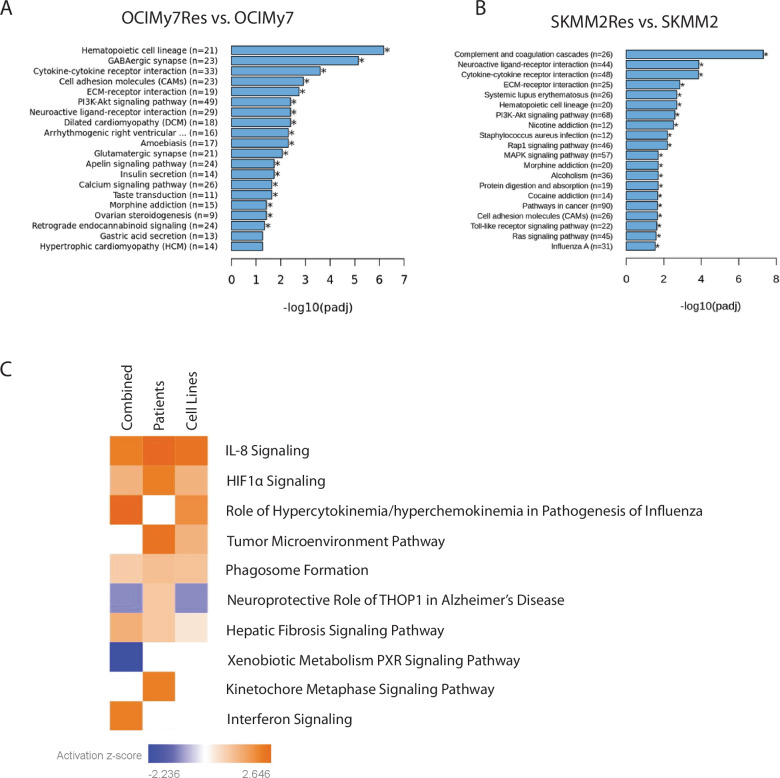
Transcriptomic analysis of VTX-sensitive and resistant samples. **A**, **B** Visualization of KEGG-based enrichment analysis of differentially expressed genes between two (OCIMy7 and SKMM2) venetoclax-resistant and sensitive cell lines. **C** Comprehensive pathways analysis contrasting all collected venetoclax-resistant cell lines (primary patient samples and human cell lines) with sensitive samples, highlighting differentially regulated pathways; orange color shows pathway activation in resistant samples, and blue shows pathway suppression.

Enrichment analysis of the differentially expressed genes from mRNA-seq revealed genes involved in cytokine-cytokine receptor interaction, cell adhesion molecules, extracellular matrix (ECM) receptor interaction, PI3K-AKT, RAS, and MAPK signaling pathways (Fig. 2A, B, Supplementary Fig. S3). We identified some common transcriptional changes shared by all resistant samples, with IPA exhibiting activation of IL-8, HIF1α, cytokine/chemokine, tumor microenvironment, and phagosome formation pathways (Supplementary Data S2, Fig. 2C). Based on the WES data from sensitive and resistant samples, we did not detect any acquired mutations in *BCL2* or other *BCL2* family members in either HMCLs or patient samples. Eight genes were shown to be commonly mutated in two resistant samples but with no clear mutational pattern (Supplementary Fig. S4).

**Fig. 3:**
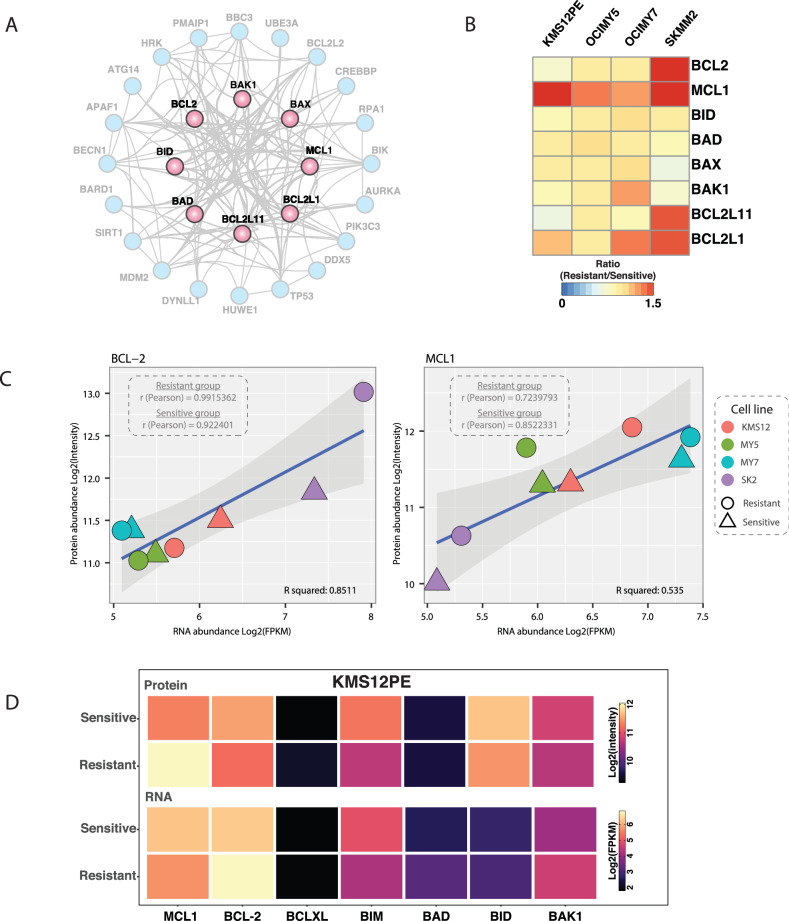
Integrated proteomic analysis of apoptosis regulation. **A** Network view (STRING-DB) of core apoptosis-related genes along with their first-degree neighbors. **B** Proteomic analysis of apoptosis genes in venetoclax-sensitive and resistant paired cell lines; protein intensity is scaled by row. **C** Non-linear regression analysis for correlation between transcriptomics and proteomics for two anti-apoptotic proteins. **D** Overview of transcriptomic and proteomic quantification of BCL-2 family proteins in parental and venetoclax-resistant KMS12PE cell lines.

VTX-sensitive and resistant cell lines were analyzed by unbiased global proteomic analysis, which identified over 9000 unique proteins (Supplementary Data S3). MCL-1 upregulation was observed in all acquired resistance cell lines in agreement with the western blot results. In addition, upregulation of BCL-XL and downregulation of BCL-2 and BIM were also observed in resistant cell lines (Fig. 3A, B).

The correlation between transcriptome and proteome data was found to be protein-specific. A strong correlation between RNAseq and protein expression was seen for BCL-2 (Fig. 3C). In contrast, MCL-1 demonstrated a weaker correlation, consistent with the upregulation observed through western blot analysis, which was not reflected in the transcriptomic data (Fig. 3C, D). Some common changes outside BCL2 family were also identified (Supplementary Data S3), such as upregulation of ISG15 and downregulation of LAIR1 were identified in at least three resistant cell lines.

### Acquired resistance to VTX can be overcome by overexpression of BIM and co-treatment with selective MCL-1 and/or BCLXL inhibitors

**Fig. 4:**
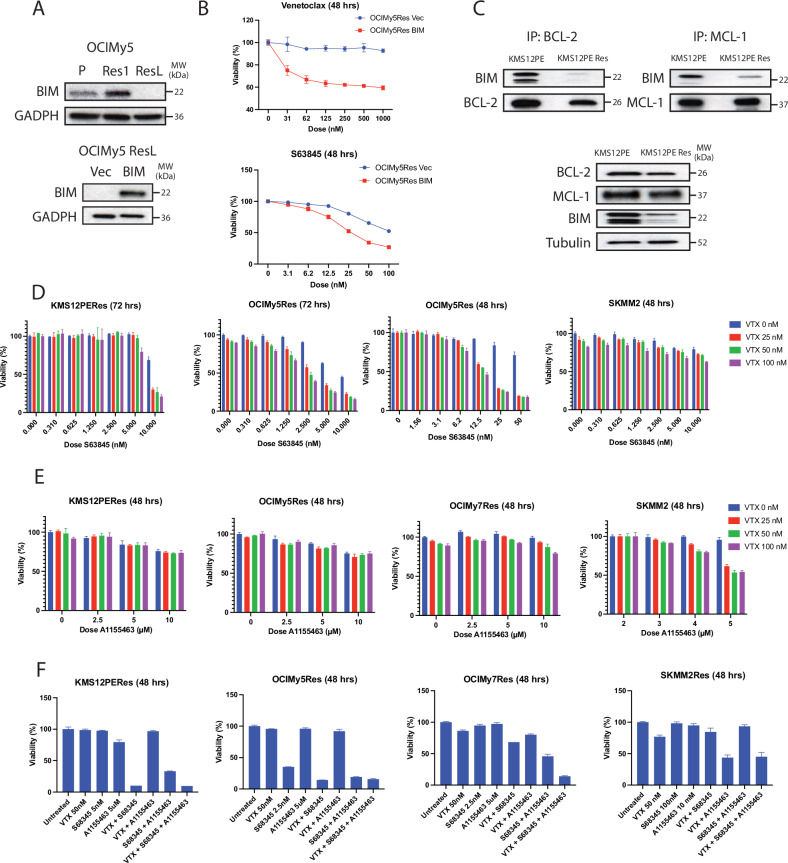
Role of BIM expression and MCL-1 inhibition in VTX sensitivity. **A** Western Blot analysis demonstrating loss of BIM expression in OCIMy5 exposed to long-term venetoclax (top panel) and re-introduction of BIM (bottom panel) for sensitivity studies. **B** MTT assay results after 48-hour incubation of venetoclax or S53845, normalized to untreated control; re-introduction of BIM lead to increased sensitivity to both compounds. **C** Immunoprecipitation assay showing decreased BIM binding to both BCL-2 and MCL-1 in KMS12Res in comparison to its sensitive counterpart. **D**–**F** MTT assays normalized to untreated control, showing activity of venetoclax plus S63845 (**D**), venetoclax plus A1155463 (**E**) and venetoclax, S63845 and A1155463 in venetoclax-resistant cell lines.

As downregulation of BIM and upregulation of MCL-1 and BCLXL are the most common changes, we assessed the contribution of each to VTX resistance. BIM expression was lost in OCIMY5Res after long-term exposure to VTX. The introduction of exogenous BIM into this cell line helped it regain response to VTX and enhanced its sensitivity to a selective BH3 mimetic targeting MCL-1, S68345 (Fig. 4A, B). In KMS12PERes, where BIM is downregulated, co-IP assay showed binding of BIM to both BCL-2 and MCL-1 was significantly decreased compared with its isogenic sensitive cell line (Fig. 4C). Overall, these findings suggest that BIM downregulation is associated with acquired VTX resistance in HMCLs.

Upregulation of MCL-1 was seen in three resistant HMCLs, but only OCIMY7Res showed an increased sensitivity to MCL-1 inhibitor, S68345 (Supplementary Fig. S5). Co-treatment with VTX and S68345 induced a potent synergistic antimyeloma activity in both OCIMY5Res and OCIMY7Res, modest synergy in KMS12PERes, as well as in other intrinsically resistant HMCLs, including JJN3, L363, KMS28 and OPM2 (Fig. 4D and Supplementary Fig. S6). High killing potency was achieved at relatively low doses for each compound. KMS11 and SKMM2 were the only HMCLs that did not exhibit strong synergistic activity with the combination. A similar synergistic activity by a combination of VTX and S68345 was also obtained in primary MM cells (Supplementary Fig. S7).

We further investigated the effects of a specific BH3 mimetic targeting BCL-XL, A1155463. Co-treatment with VTX and A1155463 only generated significant synergistic cytotoxicity in SKMM2Res, which has no MCL-1 but BCL-XL upregulation (Fig. 4E). Combination of A155463 with S68345 was shown to induce synergetic activity in three resistant HMCLs. The most potent anti-MM activity was seen when VTX was combined with both MCL-1 and BCL-XL inhibitors (Fig. 4F).

### Targeting MCL-1 post-translational modifications marginally enhances VTX sensitivity in HMCLs

Upregulation of MCL-1 protein in resistant cell lines was well not correlated with transcriptional expression, implying that post-translational mechanisms could be involved. Previous research has suggested that acetylation and phosphorylation may lead to increased MCL-1 stability and therefore, we investigated whether these post-translational modifications affected MCL-1 stability and VTX sensitivity in HMCLs [25, 26].

**Fig. 5:**
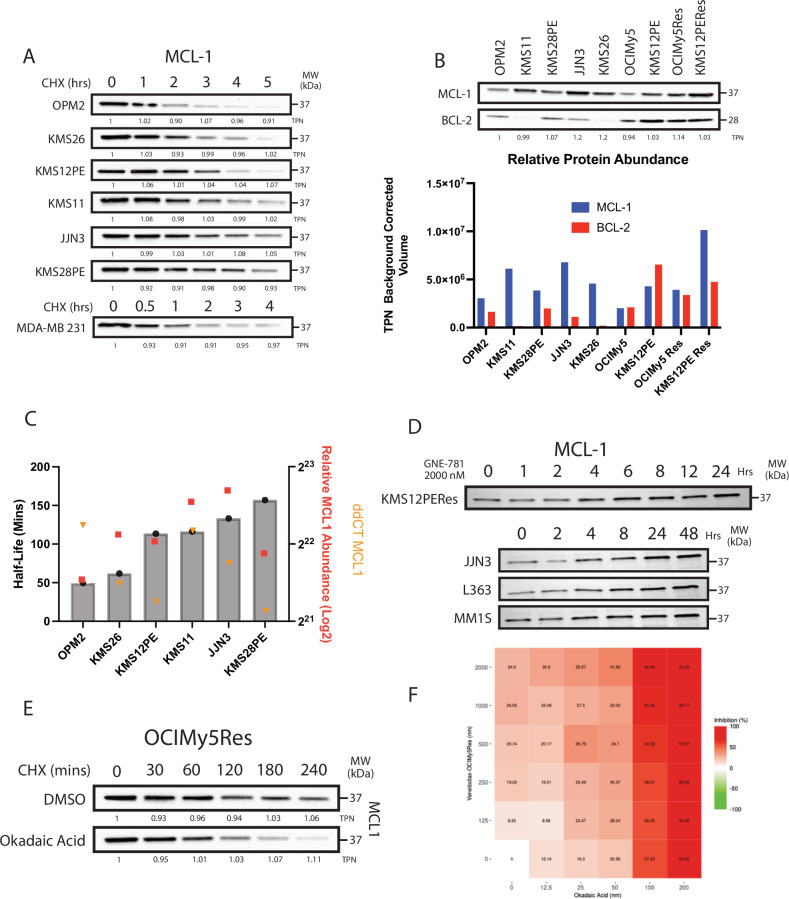
Insights into MCL-1 dynamics and impact on VTX sensitivity. **A** MCL-1 half-life quantification through Western Blot analysis following cycloheximide treatment; total protein normalization factor is labeled below each image. **B** Western Blots of BCL-2 and MCL-1 across various HMCLs with a representative bar graph of TPN background corrected volume. **C** Graphs showing MCL-1 half-life calculated using a one-phase decay analysis based on total protein normalized (TPN) local background corrected volume from Western Blot analysis. **D** Western Blot of MCL-1 quantification in various HMCLs after treatment with GNE-781 followed by cycloheximide treatment for pre-established timepoints. **E** MCL-1 quantification at different timepoints following treatment with either DMSO or okadaic acid with subsequent cycloheximide administration. **F** CellTiter-Glo-derived dose-response matrix of OCIMy5Res treated with venetoclax (left) and okadaic acid (bottom) for 24 h.

We found that MCL-1 had a significantly prolonged half-life in HMCLs when compared to that referenced in the literature, and other cancerous cell lines [25, 26] (Fig. 5A). MCL-1 protein abundance varied among HMCLs, with abundance being half-life dependent in one while transcript-dependent in others (Fig. 5B, C). We observed a trend in which cell lines with shorter MCL-1 half-lives were more transcript-dependent.

Next, we explored if inhibition of acetylation affects MCL-1 half-life and improves VTX sensitivity. A-485 or GNE-781, both p300/CBP inhibitors demonstrated significant antimyeloma activity as single agents [25, 27, 28] (Supplementary Fig. S8). However, contrary to previous studies demonstrating the downregulation of MCL-1 following treatment with A-485, both drug treatments led to MCL-1 upregulation in HMCLs (Supplementary Fig. S9). This was more evident in time-series analysis, where MCL-1 was elevated within the first few hours of treatment (Fig. 5D). Treatment with p300 inhibitors also did not significantly affect protein half-life in HMCLs and consequently, only low synergistic activity with VTX was observed (Supplementary Figs. S10 and S11).

USP9X, a deubiquitinase, is also implicated in the regulation of MCL-1 stability [29, 30].

WP-1130, a USP9X inhibitor, exhibited significant antimyeloma activity at low doses across numerous VTX-resistant HCMLs. However, treatment with WP-1130 also led to an upregulation of MCL-1 in VTX-resistant cell lines and exhibited little synergistic activity with VTX (Supplementary Figs. S12 and S13). Intriguingly, in other cell lines the pattern was more inconsistent with downregulation seen in some VTX-sensitive cell lines following treatment with WP-1130 (Supplementary Fig. 14).

Phosphorylation has been reported to also influence MCL-1 degradation, specially through protein phosphatase 2 A (PP2A), which is known to enhance MCL-1 stability [31]. We investigated the use of OA, a PP2A phosphatase inhibitor, in HCMLs with the aim of sensitizing cells to VTX. Treatment of acquired resistance HCMLs led to an average 50% reduction of MCL-1 half-life compared to DMSO treated replicates (OCIMY5Res 178.1 mins to 92.24 mins; Fig. 5E). Addition of OA to VTX treatment was associated with moderate synergistic activity in OCIMY5RES, KMS12RES and JJN3 (Fig. 5F; Supplemental Fig. S15). We also examined the combination of VTX with conventional antimyeloma drugs. Single-agent lenalidomide and bortezomib exhibited moderate antimyeloma activity across resistant cell lines (Supplemental Fig. S16). However, combining these agents with VTX did not result in significant synergistic activity in most of the cell lines.

### Targeting PI3K and RTK enhanced VTX activity via downregulation of MCL1 and BCLXL, and upregulation of BIM

**Fig. 6:**
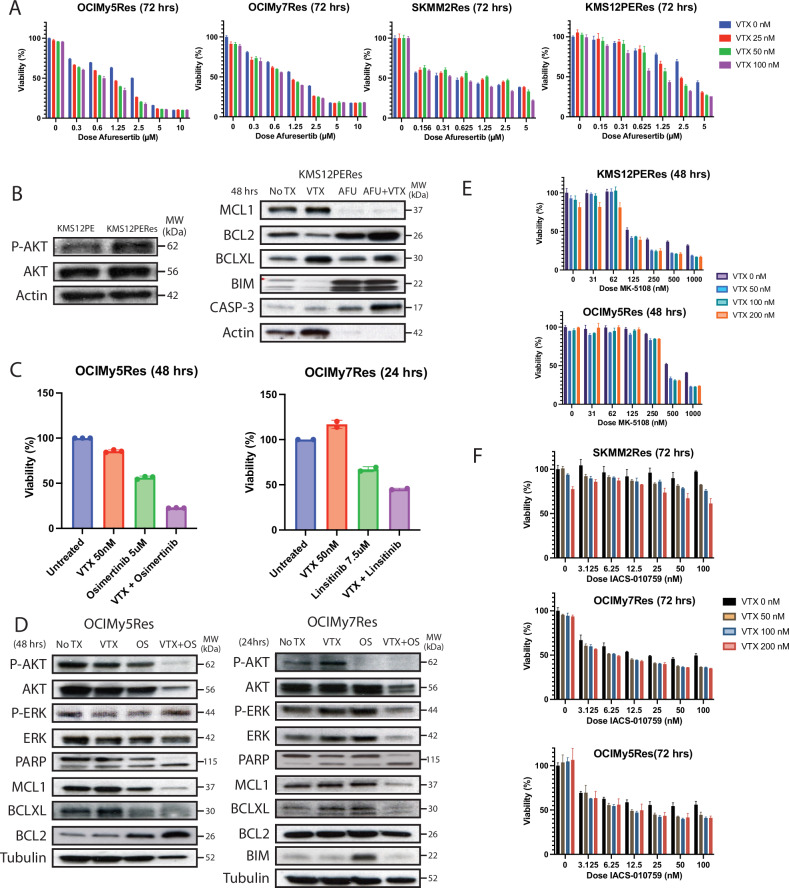
Synergistic effects of tyrosine kinase inhibitors on VTX sensitivity. **A** MTT assays of four venetoclax-resistant cell lines treated with venetoclax in combination with afuresertib for 72 h.; viability is normalized to untreated control. **B** Western Blot analysis of paired KMS12PE and KMS12Res cell lines, and with apoptotic protein quantification in KMS12PERes following treatment with venetoclax and/or afuresertib. **C** MTT assay of OCIMy5Res treated with venetoclax and/or osimertinib and OCIMy7Res treated with venetoclax and/or linsitinib for 72 h.; viability is normalized to untreated control. **D** Western Blot analysis of OCIMy5Res and OCIMy7Res proteins following treatment with osimertinib (48 h.) and linsitinib (24 h), respectively. **E**, **F** MTT assays of cell lines treated with venetoclax in combination with MK-5108 (**E**) or IACS-010759 (**F**); viability is normalized to untreated control.

Pathway analysis of differentially expressed genes between isogenic sensitive and resistant HMCLs revealed enrichments in PI3K/AKT and MAPK pathways. Therefore, we investigated the effects of inhibition of PI3K and MAPK inhibition on VTX sensitivity in resistant HMCLs. Inhibition of AKT using afuresertib increased VTX sensitivity in all resistant HMCLs (Fig. 6A). In contrast, MAPK inhibition with cobimetinib only exerted subtle effects on VTX sensitivity (data not shown). Through western blot analysis, we showed that AKT inhibition in KMS12PERes resulted in the downregulation of MCL-1 and BCL-XL and the upregulation of BIM (Fig. 6B).

To identify upstream changes regulating PI3K activity and BCL-2 family member expression, we focused on upregulated genes in resistant samples. We observed upregulation of several cytokines (such as IL-32), growth factors (including EGF/EGF-like, FGF, and IGF1/IGF-like), or their receptors (Supplementary Data S4). By co-treating resistant HMCLs with VTX and selective inhibitors targeting IGF1, FGF, and EGF receptor-associated tyrosine kinases (RTKs), we demonstrated that RTK inhibition of FGF, EGF, and IGF-sensitized VTX activity, particularly in resistant cell lines exhibiting upregulated FGF, EGF/EGF-like, IGF1/IGF-like gene expression (Fig. 6C; Supplemental Fig. S17). Inhibition of EGF and IGF receptor activity in OCIMY5Res and OCIMY7Res cells induced upregulation of BIM or downregulation MCL-1 and BCL-XL (Fig. 6D). Combination of VTX with these inhibitors was shown to generate synergistic anti-MM cytotoxicity.

### Inhibition of Aurora kinase A (AURKA) and ETC1 increase sensitivity of resistant HMCLs to VTX

Aura kinase A (AURKA) plays essential roles in regulating cell division during mitosis and serves as a regulator for several signaling pathways, including PI3K/Akt, mTOR, β-catenin/Wnt and NF-κB pathways [32, 33]. AURKA expression was elevated in both resistant cell lines and relapsed patient samples. Combination of VTX and a selective AURKA inhibitor, MK-5108, resulted in synergistic activity in two resistant HMCLs with elevated AURKA (Fig. 6E).

Recent studies have demonstrated that respiratory electron transport chain (ETC) activity can serve as a predictor for response to VTX in MM [34]. Several ETC gene expression levels were altered (mRNA-Seq data) in resistant samples, especially in samples from relapsed patients. However, co-treatment with VTX and IACS-010759, a mitochondrial complex I inhibitor, only slightly enhanced VTX (Fig. 6F).

## Discussion

We have comprehensively examined the molecular mechanisms of both intrinsic and acquired VTX resistance in MM, highlighting the complex interplay of BCL-2 family proteins with various signaling pathways. Consistent with previous reports, our study confirms the role of BCL-2 family regulation in VTX resistance. Specifically, downregulation of BIM and upregulation of MCL-1 and BCL-XL were identified as the most common changes across resistant samples. VTX has BIM-dependent and BIM-independent mechanisms of action, which partially explains why BIM downregulation is observed in resistant cell lines [9, 35, 36]. Our study demonstrated that reintroducing BIM into resistant cells not only partially restores VTX sensitivity but could also enhances response to selective MCL-1 inhibitors, since MCL-1 is most likely responsible for sequestering BIM and ultimately preventing it from activating the pro-apoptotic proteins BAX and BAK. By reintroducing BIM, we overcome the neutralizing capacity of MCL-1, re-sensitizing cells to VTX. Therefore, targeting BIM or its regulatory pathway provides a promising therapeutic approach. Furthermore, the downregulation of other pro-apoptotic proteins such as BIK, NOXA, and PUMA also likely contribute to resistance. NOXA, in particular, is known to facilitate the degradation of MCL-1 by recruiting the E3 ligase MULE/HUWE1 which disrupts its interaction with the deubiquitinase USP9X [37]. The loss of NOXA could, therefore, lead to increased MCL-1 stability and accumulation, enhancing the cell’s anti-apoptotic capacity. We indirectly studied this interaction by using a USPX inhibitor. This aligns with our observation that MCL-1 protein levels increase without concurrent transcriptional upregulation. Further investigating the roles of these BH3-only proteins through additional knockout studies could provide deeper insights into their contributions to resistance mechanisms.

The increased expression of BCL-XL and MCL-1 in resistant cell lines raises important questions about the potential alterations in protein complex formation contributing to resistance. Previous studies have demonstrated that BIM binding can stabilize MCL-1. A shift in BIM binding from BCL-2 and MCL-1 could potentially lead to increased MCL-1 stability [38]. Our co-immunoprecipitation experiments in a single cell line showed decreased BIM binding to both BCL-2 and MCL-1, likely a consequence of decreased overall expression in resistant cell lines. Nevertheless, the amount of BIM co-immunoprecipitated with MCL-1 was higher than with BCL-2, implying that despite the decreased levels of BIM, its preferential binding to MCL-1 could contribute to increased stability and, thus, resistance. Beyond their BH3-only and pro-apoptotic effecter sequestering role, the increased BCL-XL and MCL-1 expression likely also contribute to resistance through their influence on metabolic pathways, leading to adaptations supporting survival under therapeutic stress along through interaction with signaling pathways impacting cell cycle progression and DNA damage response mechanisms. Expanding co-immunoprecipitation experiments to other resistant cells and performing BH3 profiling could provide further insights into the functional consequence of these expression changes.

Despite the upregulation of MCL-1 and BCL-XL detected in most of our resistant cell lines, which potentially shifts the cell’s dependence from BCL-2 and creating a therapeutic window, co-treatment with VTX and a BCL-XL showed minimal synergistic activity in most cases. In contrast, co-treatment with VTX and MCL-1 inhibitors, or a combination of BCL-XL and MCL-1 inhibitors provided a more effective approach. This suggests that MCL-1 plays a critical part in the context of VTX resistance in most cases, possibly justified by the superior binding affinity of MCL-1 [39–41]. We also noted that despite a consistent upregulation of MCL-1 in resistant cell lines, there was no clear correlation between MCL-1 levels and response to MCL-1 inhibition. This could be explained by functional redundancy, where other anti-apoptotic proteins compensate for the inhibition of MCL-1 [42].

Previous clinical trials, such as BELLINI and CANOVA, have demonstrated that combining VTX with standard myeloma therapies can be effective in some patients with relapsed or refractory myeloma [8, 43]. Dexamethasone has been reported to increase BIM expression and promote BCL-2 priming [44]. Treatment with proteosome inhibitors has been shown to stabilize and upregulate NOXA [45, 46]. We tested a combination of VTX with conventional antimyeloma therapeutics. However, combining these drugs with VTX demonstrated underwhelming synergistic activity in most of our resistant cell lines. The lack of synergy suggests that acquired resistance may alter apoptotic dependencies and diminish the efficacy of combinations that are effective in VTX-naive settings. Previous studies have also suggested that VTX resistance may confer cross-resistance to other standard-of-care antimyeloma drugs [47]. This underscores the potential limitations of combining VTX with currently approved MM regimens in VTX-relapsed patients.

We found that the highest anti-MM activity was achieved in resistant cell lines when VTX was combined with both MCL-1 and BCL-XL inhibitors, suggesting that even reduced BCL-2 is still significantly involved in survival, and BCL-XL and MCL-1 upregulation cannot sufficiently offset its loss or reduced function. Therefore, to counter resistance, alternating therapies that continuously target the shifted myeloma cell apoptotic dependencies might be effective.

While our study demonstrated that co-treatment with MCL-1 inhibitors can effectively overcome acquired resistance, we acknowledge the clinical challenges associated with this combination due to toxicity concerns, particularly cardiotoxicity linked to earlier-generation MCL-1 inhibitors [48]. Our findings suggest that the significant degree of synergy achieved with the combination raises the possibility of reducing dosing of each compound, potentially mitigating toxicity while retaining efficacy. Moreover, recent advancements have introduced new MCL-1 inhibitors with improved safety profiles which hopefully renews the potential for combining MCL-1 inhibitors with VTX in clinical settings [49]. Efforts to evaluate optimizing dosing strategies may help provide a viable therapeutic option for this subgroup of patients.

Given MM’s pronounced reliance on MCL-1, we explored whether upregulation of MCL-1 in VTX-resistant cell lines is associated with MCL-1 stabilization. Shimizu et al. identified a mechanism in which MCL-1 stability is increased in breast and prostate cancer cell lines through acetylation by p300, which enhances the de-ubiquitinating function of USP9X, facilitating apoptosis evasion [25]. To investigate this, we treated HMCLs with p300 and USP9X inhibitors. Contrary to expectations, treatment upregulated MCL-1, and only limited synergistic activity was observed in combination with VTX. Both p300 and USP9X inhibitors exhibited strong antimyeloma activity as monotherapy, suggesting they might work via alternative pathways. The observed MCL-1 upregulation may be due to a compensatory survival mechanism. PP2A is an MCL-1 stabilizing phosphatase, and its inhibition has also been linked to a reduction in MCL-1 half-life in MM [31]. We found that PP2A inhibition with OA did result in a reduction of MCL-1 half-life in two VTX-resistant HMCLs, but was associated with only modest, albeit noteworthy, synergistic activity. Despite this reduction, MCL-1’s half-life in MM cell lines remained longer than in other malignancies. These findings suggest that while post-translational modifications can influence MCL-1 stability and VTX sensitivity, outcomes are contingent on specific cellular environment and modification nature. Given that all of these inhibitors share a molecular pathway for MCL-1 stability centered on ubiquitin modulation, it is plausible that MCL-1 stability in MM is not ubiquitin-dependent [26].

Moreover, the overexpression of BCL-XL and MCL-1 may contribute to a chemo-resistant phenotype extending beyond VTX. This could explain the limited synergy observed when combining VTX with conventional antimyeloma agents in our resistant cell lines. The cells may have shifted their apoptotic dependence from BCL-2 to BCL-XL and MCL-1, highlighting the potential effectiveness of combination therapies targeting these proteins. Further analysis of the transcriptomic data revealed that genes involved in microenvironment interactions and interferon signaling were significantly upregulated, suggesting enhanced inflammatory response and alterations in the tumor microenvironment. The upregulation of IL-8, HIF1α, and interferon signaling all suggest mechanisms that enhance stress-induced adaptation and immune evasion.

Based on gene expression and pathway analysis data, we investigated the effects of inhibition of the pathways, i.e., PI3K/AKT, MAPK, and RTK, enriched in the differential gene expression between sensitive and resistant cell lines. Consistent with a previous study [50], AKT inhibition bolstered VTX sensitivity in all resistant HMCLs, while inhibition of specific RTKs, such as FGF, EGF, and IGF, sensitized the cells overexpressing these growth factors. We demonstrated that enhanced sensitivity was associated with BCL-2 family protein shifts, including MCL-1, BCL-XL downregulation, and BIM upregulation. RAS mutations and MAPK activation have been implicated in VTX resistance in other hematological malignancies such as AML [51]. These AML models have shown that MAPK is involved in MCL-1 stabilization and BIM inactivation, but did not observe a significant synergy between VTX and MAPK inhibitors in our resistant cell lines [52]. This suggests that MAPK signaling may not be a predominant mechanism of resistance in our models. Additionally, higher AURKA expression was also noted in several resistant samples, and its inhibition increased VTX sensitivity. Several AURKA inhibitors (AKIs) have undergone preclinical testing, with some advancing to clinical trials [32, 53–55]. Targeting these pathways, such as inhibitors of iL-8 signaling or HIF1α could potentially sensitize cells to VTX.

Anti-apoptotic proteins are known to influence mitochondrial bioenergy and metabolic pathways, leading to adaptations that support survival under therapeutic stress. Recent studies have also highlighted that MM cell lines susceptible to VTX exhibit lower ETC activity relative to inherently VTX-resistant cells [34, 56]. By analyzing RNAseq data, we observed altered expression of several ETC genes in resistant samples. While our study showed limited effects with mitochondrial complex, I inhibition, targeting metabolic vulnerabilities associated with increased BCL-XL and MCL-1 expression might offer alternative strategies. However, co-treatment of VTX with IACS-010759, an ETC complex 1 inhibitor, yielded only marginally enhanced VTX, suggesting ETC activity may not play a major role in VTX resistance.

Comparing the mechanisms of resistance seen in our models to those reported in other hematological malignancies reveals both commonalities and differences. Like myeloma, resistance in AML/CLL is often associated with the upregulation of alternative anti-apoptotic proteins, downregulation of pro-apoptotic proteins, and activation of survival signaling pathways. However, unlike in CLL, where BCL-2 mutations, such as G101V, are common, we did not detect BCL-2 mutations in our resistant samples, suggesting that genetic alterations in BCL-2 are not a common resistance mechanism [21]. The cellular and molecular contexts may also differ, such as is the case with AML, where resistance mechanisms include the emergence of monocytic subclones that rely on MCL-1, a shift driven by the differentiation state of cells [57]. In CLL, resistance also involves alternative pathways such as NF-kB. While the similarities across malignancies emphasize the central role of the BCL-2 family, the mechanisms and cellular contexts differ significantly among diseases, showcasing the complexity of optimal therapeutic implications.

In conclusion, our study offers valuable insights into the intricate mechanisms dictating VTX resistance. The confirmation of the relevance of key proteins, including MCL-1, BIM, and BCLXL, and the identification of critical pathways, including PI3K and RTK, related to VTX resistance opens potential avenues for therapeutic interventions. Our results highlight the possibility of combining VTX with MCL-1 and BCL-XL inhibitors or PI3K and RTK inhibitors for further research. Crucially, the diversity of resistance mechanisms reported demonstrates the substantial value of an in-depth evaluation of the resistance profile of primary patients, emphasizing the need for tailored strategies to overcome or prevent resistance to VTX. Continuous understanding of the molecular determinants of response to BH3 mimetics will enable the development of predictive biomarkers and help optimize treatment strategies. Future studies will focus on functional assays such as extended co-IP experiments and BH3 profiling across multiple resistant cell lines to elucidate the impact of protein complex alterations on resistance. Additional knockout studies could also provide deeper insights into BH3-only protein’s contributions to resistance.

## Supplementary information

Supplemental Figures and Tables

## Data Availability

All data relevant to the study are included in the article and/or supplementary material. The datasets generated during and/or analyzed during the current study are available from the corresponding author upon reasonable request. The mass spectrometry proteomics data have been deposited in the ProteomeXchange Consortium via the PRIDE partner repository with the dataset identifier PXD047149. (Username: reviewer_pxd047149@ebi.ac.uk; Password: GUQn6Yrk).
