# Supplementary material for: Exploring BCL2 regulation and upstream signaling transduction in venetoclax resistance in multiple myeloma: potential avenues for therapeutic intervention
Source: Blood Cancer J. 2025 Feb 5;15(1):10. doi: 10.1038/s41408-025-01215-x (PMC11799149; doi:10.1038/s41408-025-01215-x)

**Table S1. Human Myeloma Cell Lines Characteristics**

| **Cell Line Name** | **Sex** | **Ancestry** | **Canonical Translocation** | **TC Class** | **Tissue Source of Origin** |
| --- | --- | --- | --- | --- | --- |
| KMS12PE | Female | East Asia | t(11;14) | D1 | Pleural Effusion |
| OCI-My5 | Male | Europe | t(14;16) | MAF | N/A |
| OCI-My7 | Male | Europe | t(11;14) | D1 | N/A |
| SK-MM-2 | Male | Europe | t(11;14) | D1 | Peripheral Blood |
| JJN3 | Female | Europe | t(14;16) | MAF | Bone Marrow |
| KMS26 | Male | East Asia | t(4;14) | MMSET | Pleural Effusion |
| KMS11 | Female | East Asia | t(4;14)  t(14;16) | MMSET | Pleural Effusion |
| OPM2 | Female | East Asia | t(4;14) | MMSET | Peripheral Blood |
| KMS28PE | Female | East Asia | t(4;14) | MMSET | Pleural Effusion |

Antibodies against BCX-L, MCL-1, PARP, BIM, PUMA, BID, BIK, NOXA, BAK, BAX, P-AKT (Ser 473 or Thr308), AKT, p-ERK1/2, and ERK1/2 were from Cell Signaling Technology (Danvers, MA). Anti-BCL2 antibody was from Santa Cruz. Mouse antibodies against MCL-1 antibodies were from Angio-Proteomic (Boston, MA). Venetoclax (VTX), S68345, A1155463, Afuresertib, BGJ398, Linsitinib, Osimertinib, MK5108, Lenalodomide and Bortezomib were all from Selleck Chemical LLC (Houston, TX). Cyclohexamide was purchased from Sigma Alrich, WP1130 (Degrasyn) from MedChemExpress and Okadaic Acid from Enzo Life Sciences.

**Figure S1. CellTiter-Glo Assay after venetoclax exposure (24 hrs) in venetoclax resistant cell lines after no venetoclax exposure for 3 months**

**Figure S2. BCL2/BCLXL ratio in parental and VTX-resistant HMCLs based on mRNAseq data.**

**
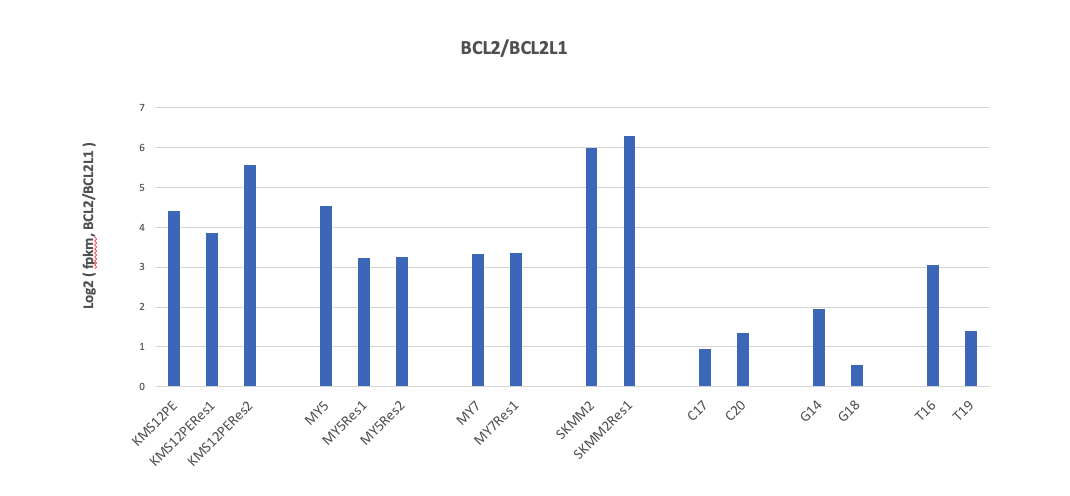
**

**FigureS3. Enrichment analysis (KEGG) of parental and venetoclax-resistant HMCLs**


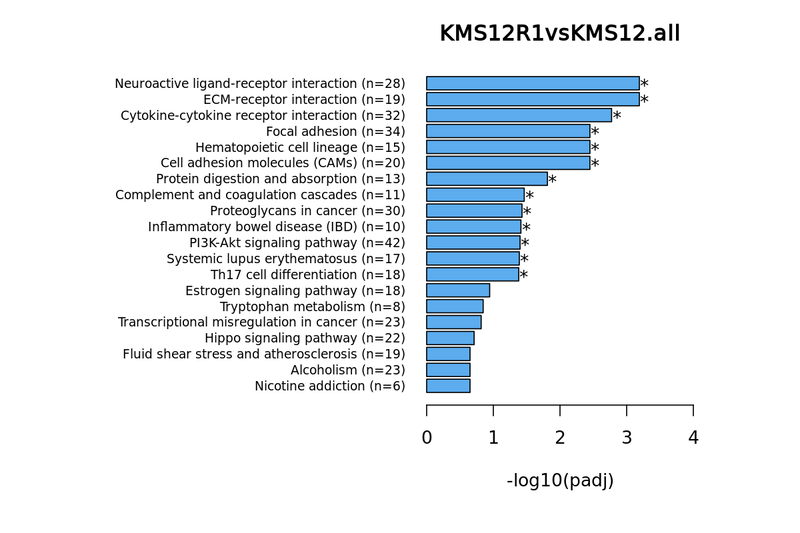


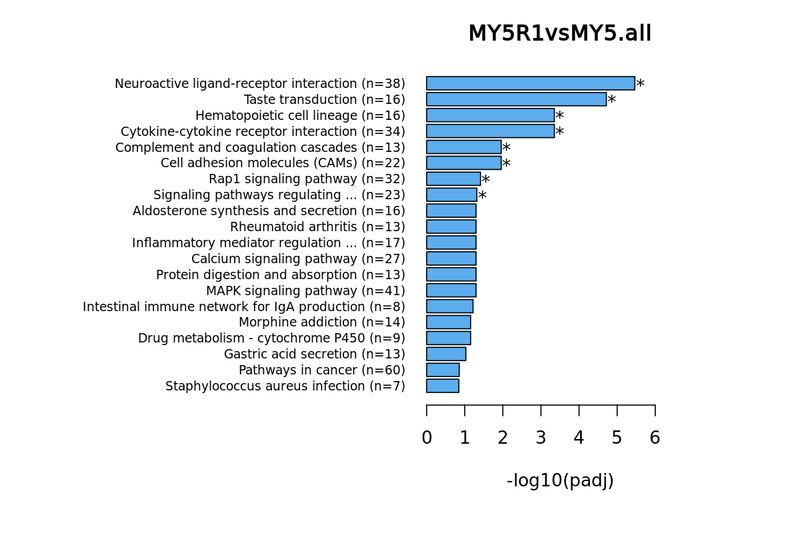


**Figure S4. Identified mutations in primary venetoclax-resistant patient samples.**


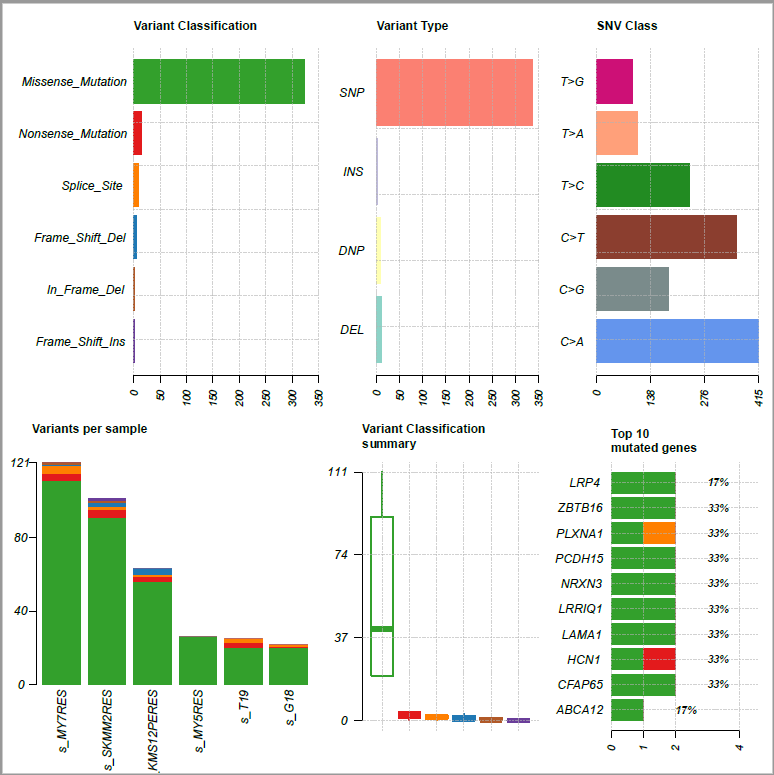


**Figure S5.** **MCL-1 inhibition sensitivity in venetoclax-resistant MM cell lines**


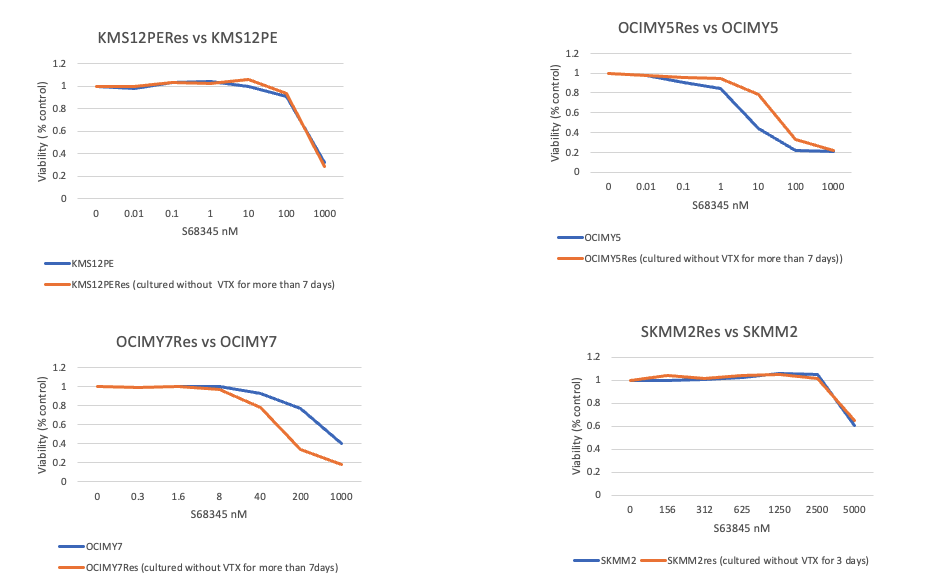


**Figure S6. Dual BCL-2 and MCL-1 inhibition (24 hrs) leads to strong synergistic activity in venetoclax-resistant cell lines.**


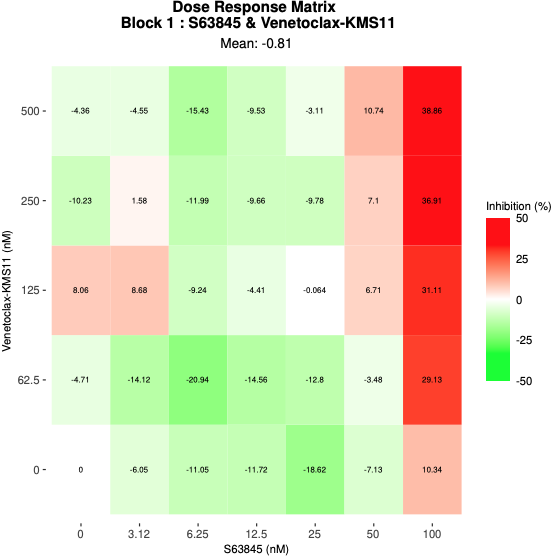

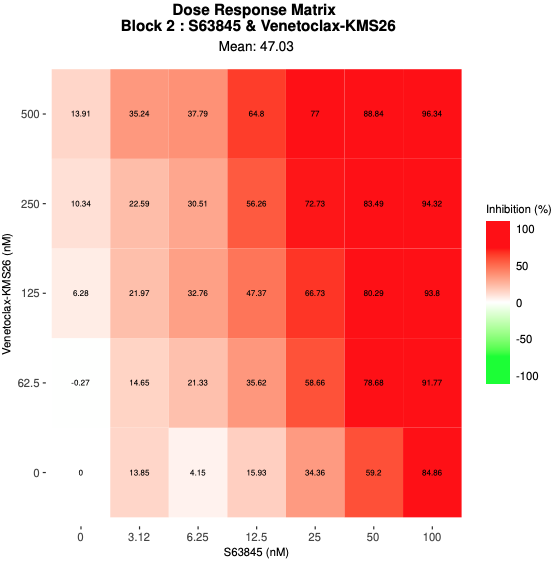


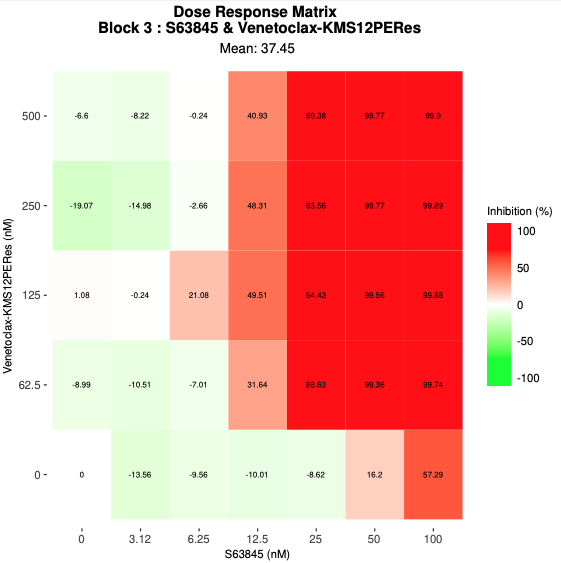

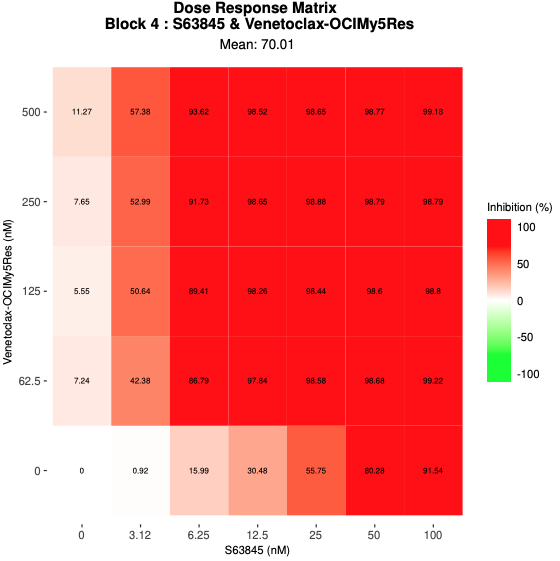


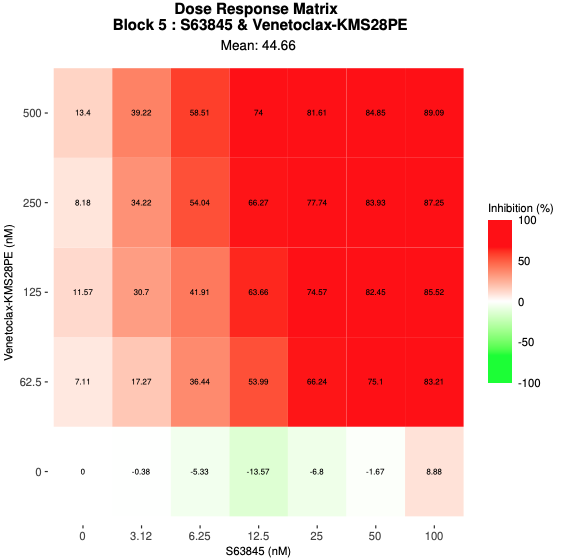

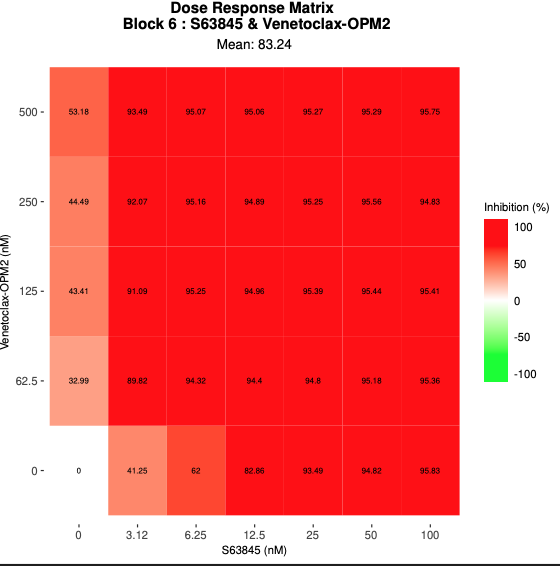


**Figure S7. Dual BCL-2 and MCL-1 targeting in primary patient samples**

**
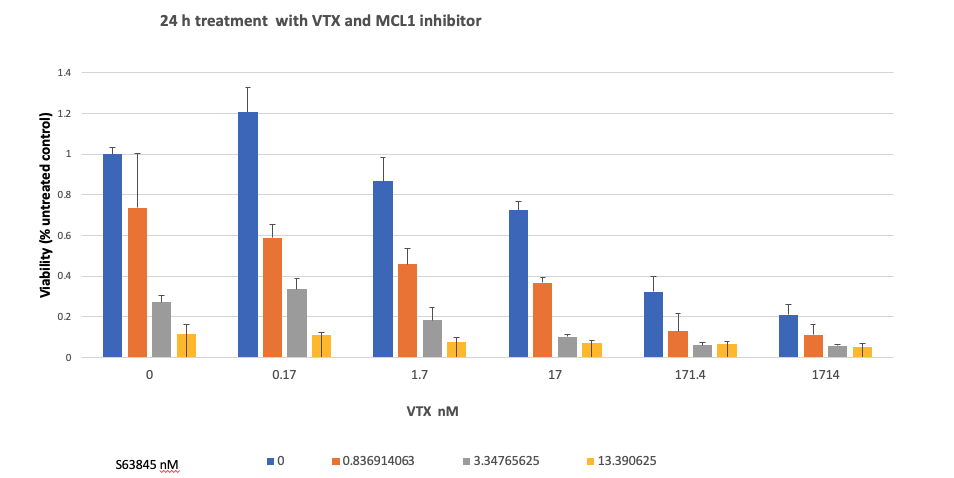
**

**Figure S8.** **CellTiter-Glo Assays after incubation of HMCLs with single agent A-485 or GNE-781 for 72 hrs.**

**Figure S9. Western-Blot analysis of MCL-1 levels following p300 inhibition in HMCLs**


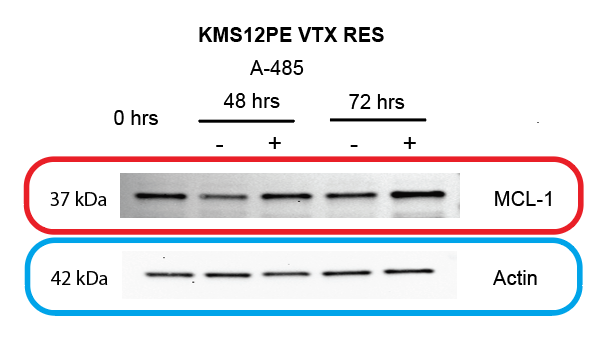

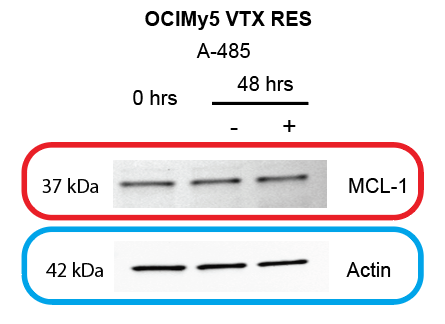


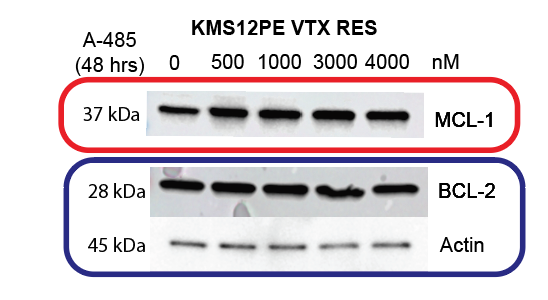

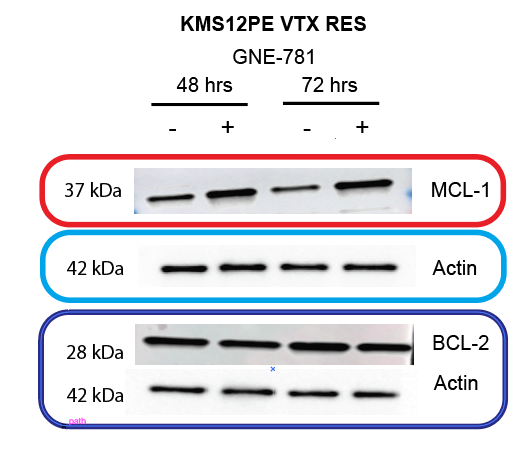


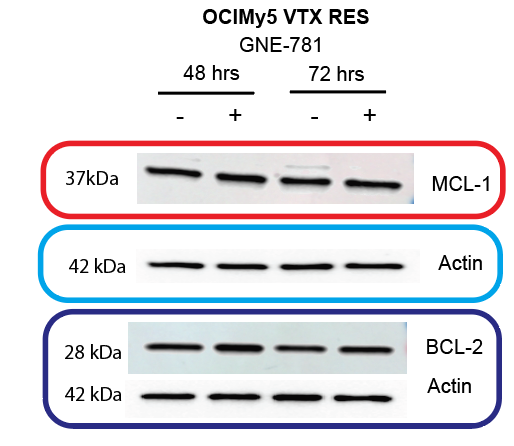
 **
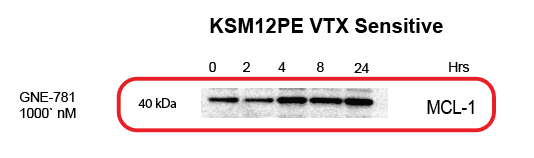
**

**Figure S10. Western Blot analysis of MCL-1 half-life following p300 inhibition with either A-485 or GNE-781 for 24 hrs.**

**
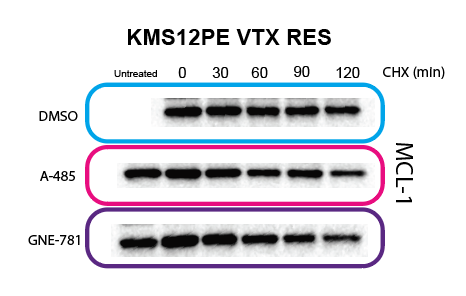

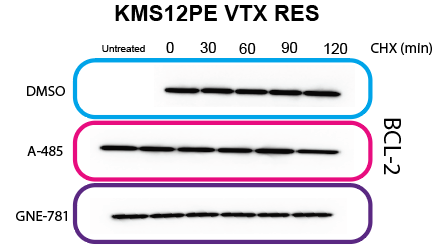
**

**
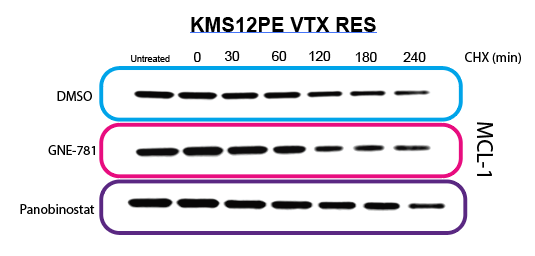

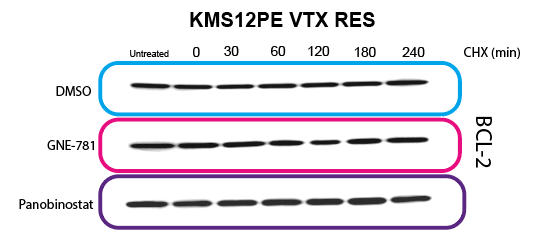
**

**Figure S11. Synergy assays of venetoclax plus p300 inhibition with either A-485 (24 hrs) or GNE-781 (72 hrs)**

**KMS12PERes**


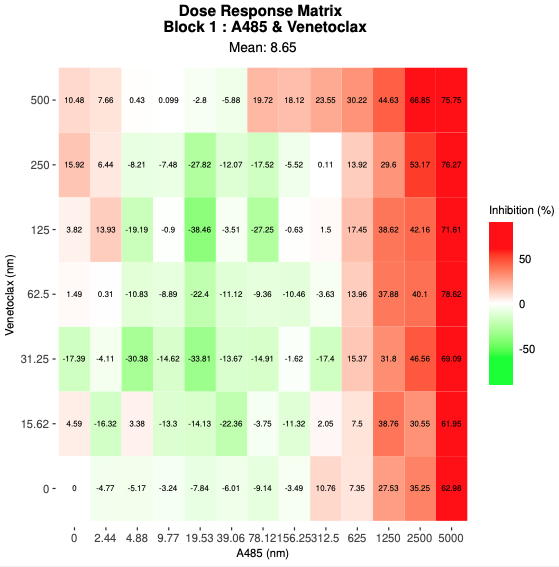

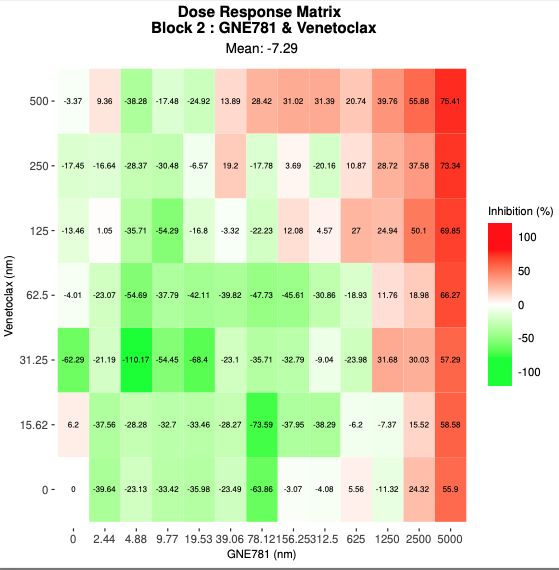


**Figure S12. Western Blot analysis of MCL-1 levels following treatment with WP-1130 in KMS12PERes and OCIMy5Res**

**
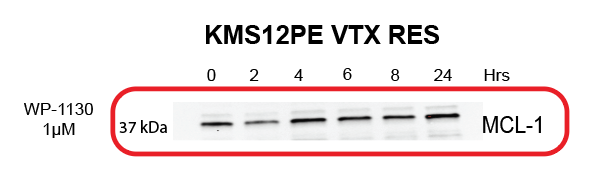
**

**
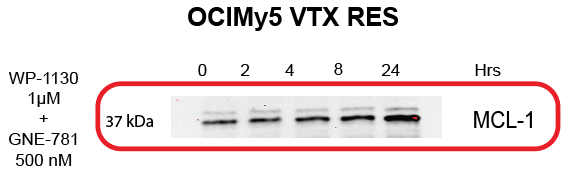
**

**Figure S13. CellTiter-Glo assays following treatment with WP-1130 for 24 hrs.**

**OCIMy5Res KMS12PERes**

**
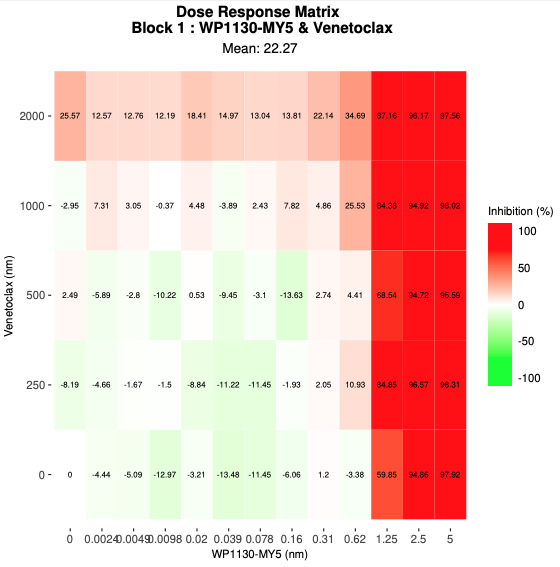

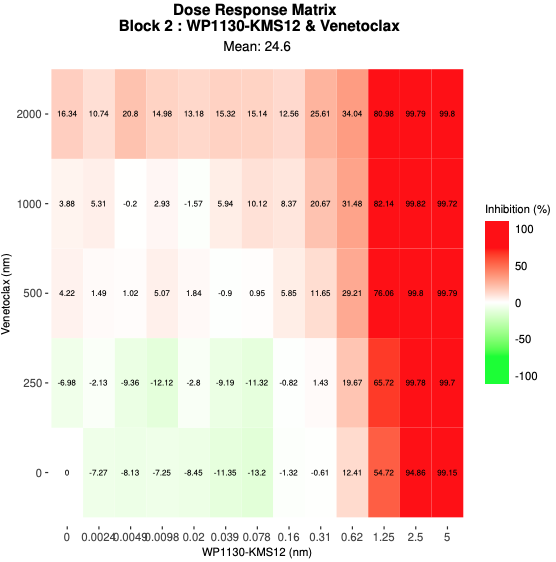
**

**KMS11 KMS26**

**
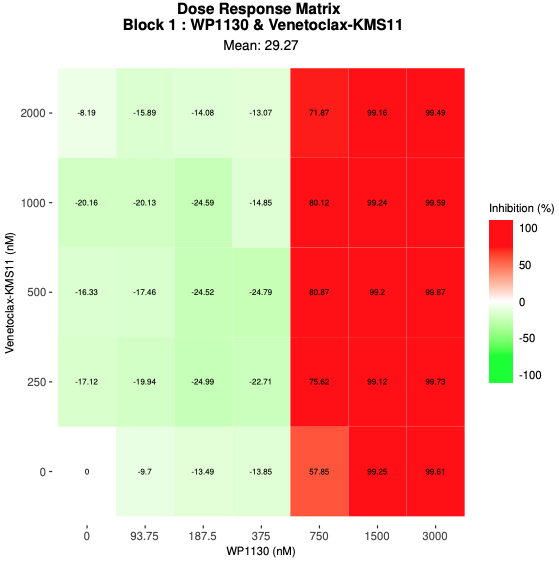

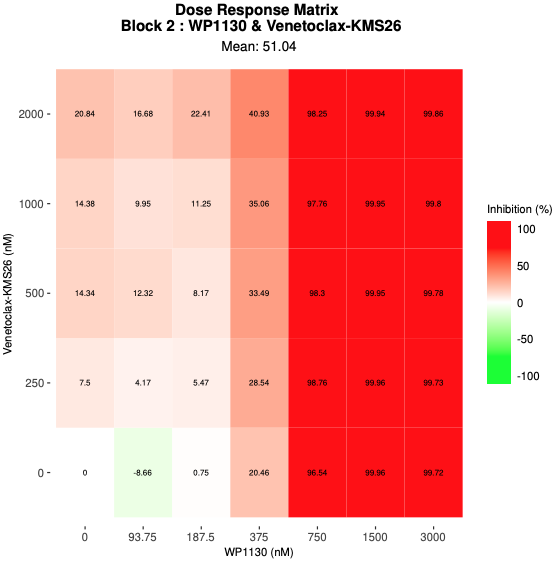
**

**KMS28 OPM2**

**
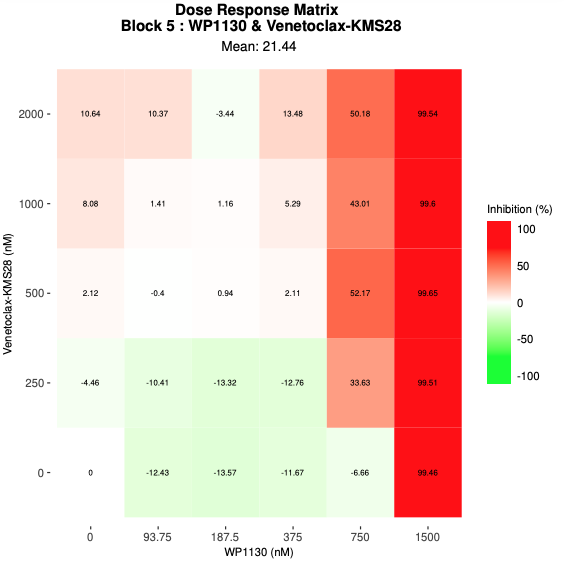

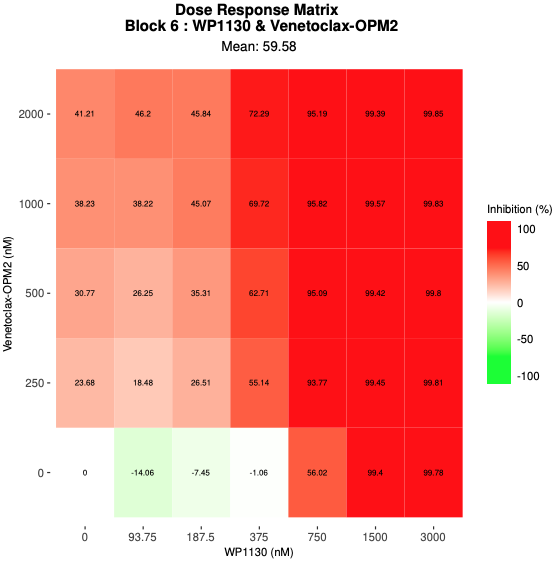
**

**Figure S14. Western Blot analysis of MCL-1 levels following treatment with WP-1130**

**
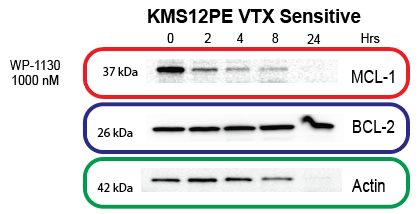
**

**
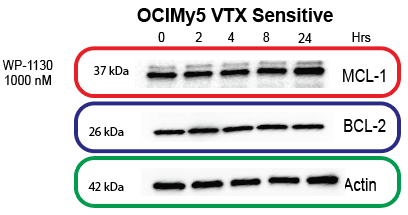
**


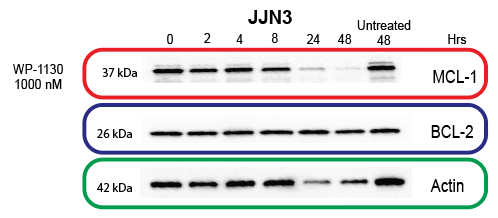


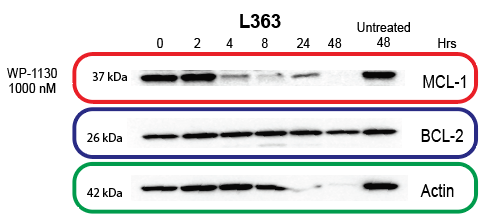


**Figure S15. Doses response matrixes from synergy assays assessing venetoclax in combination with okadaic acid.**

**JJN3**


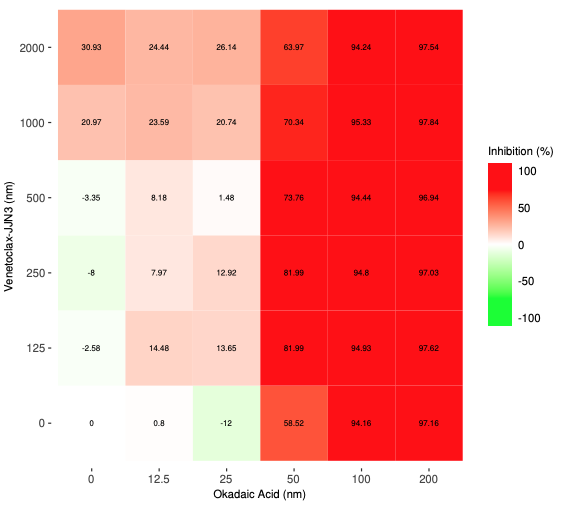


**KMS26**


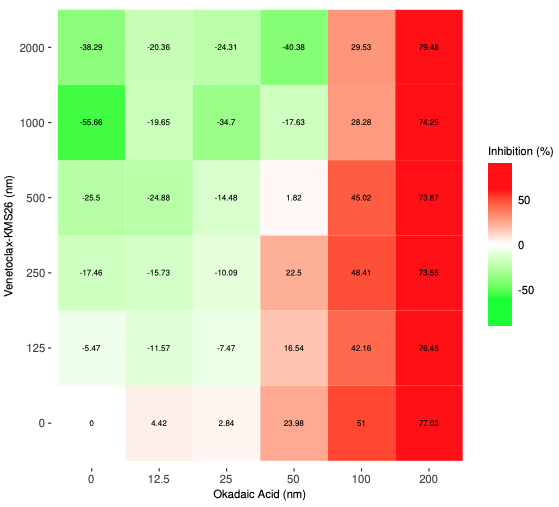


**KMS12PERes**

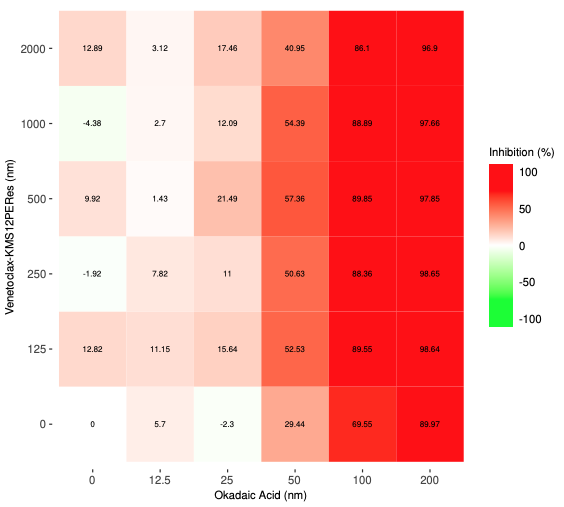


**Figure S16. CellTiter Glo synergy assays following treatment with venetoclax in combination with conventional anti-myeloma drugs (bortezomib, dexamethasone and lenalidomide)**


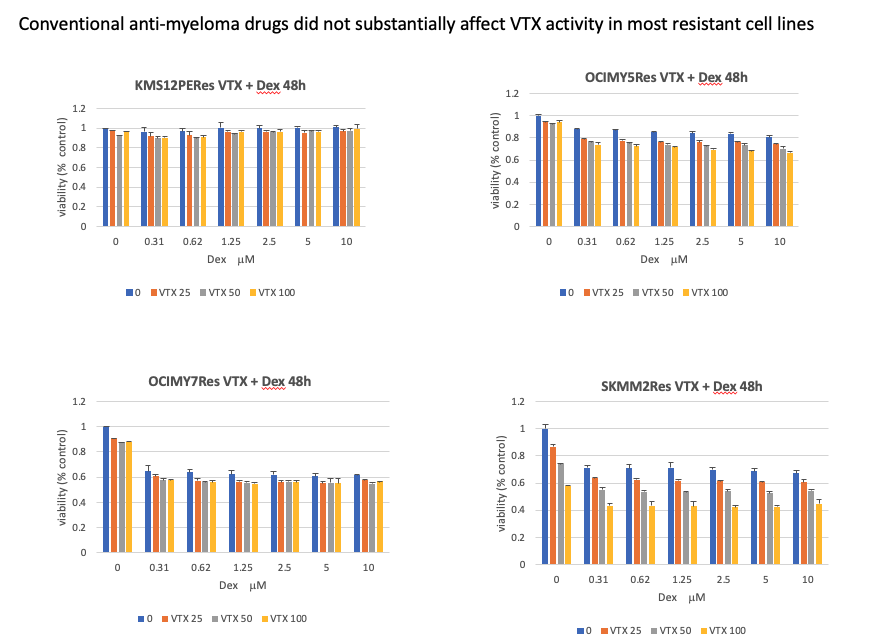


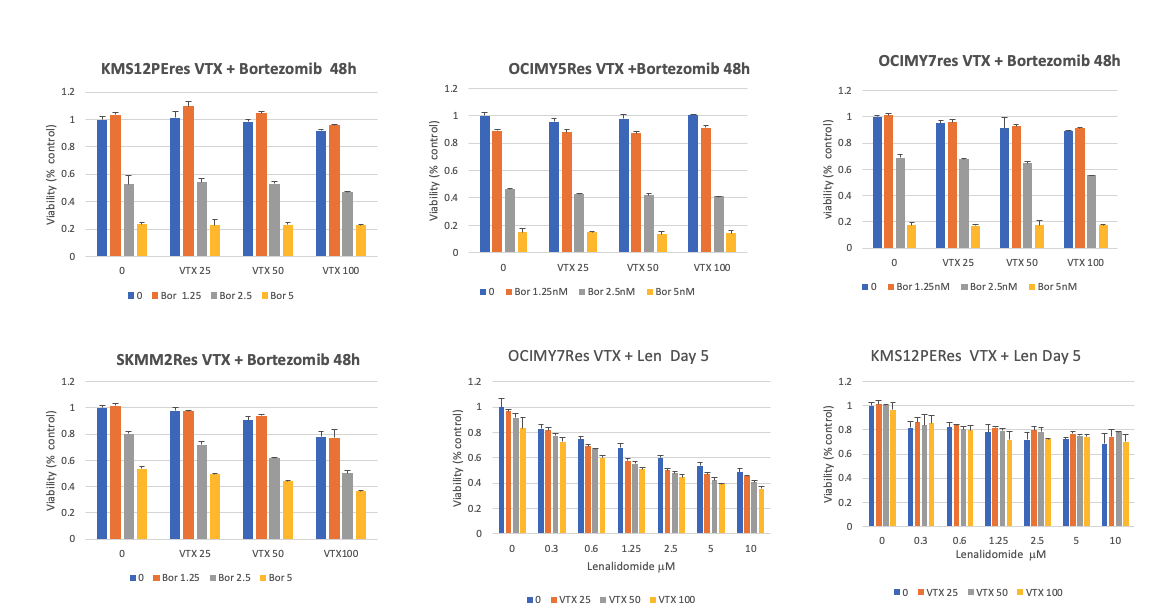


**Figure S17. MTT Assays of RTK inhibition in venetoclax-resistant cell lines**


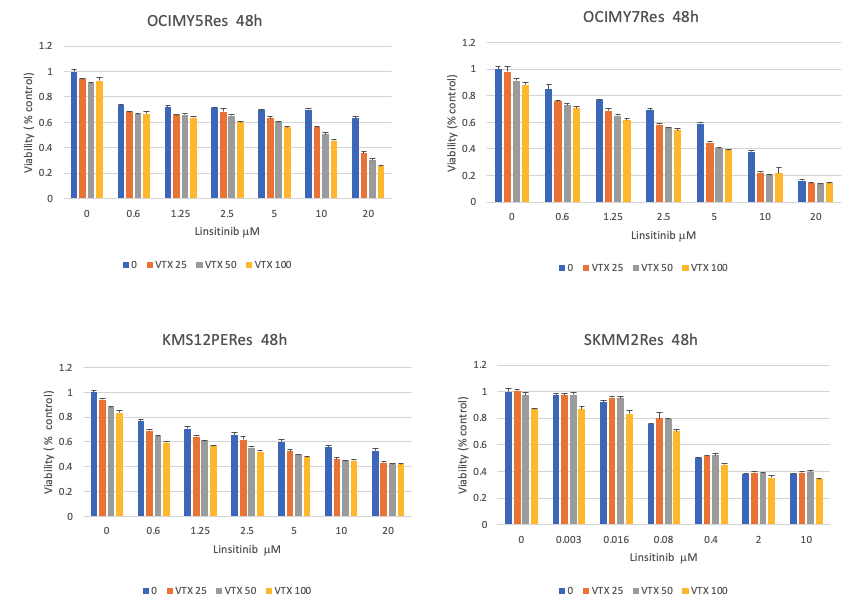


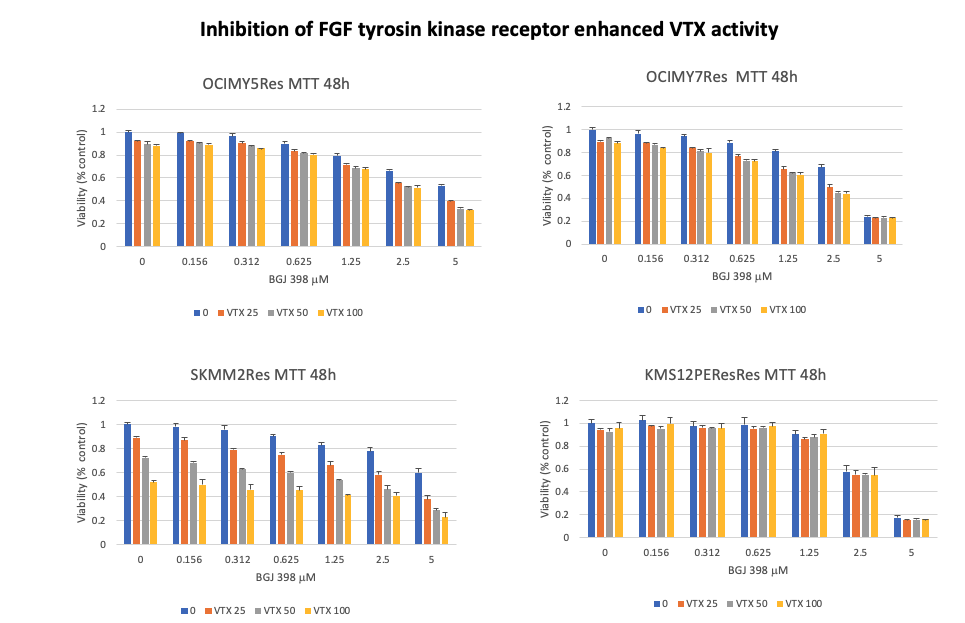


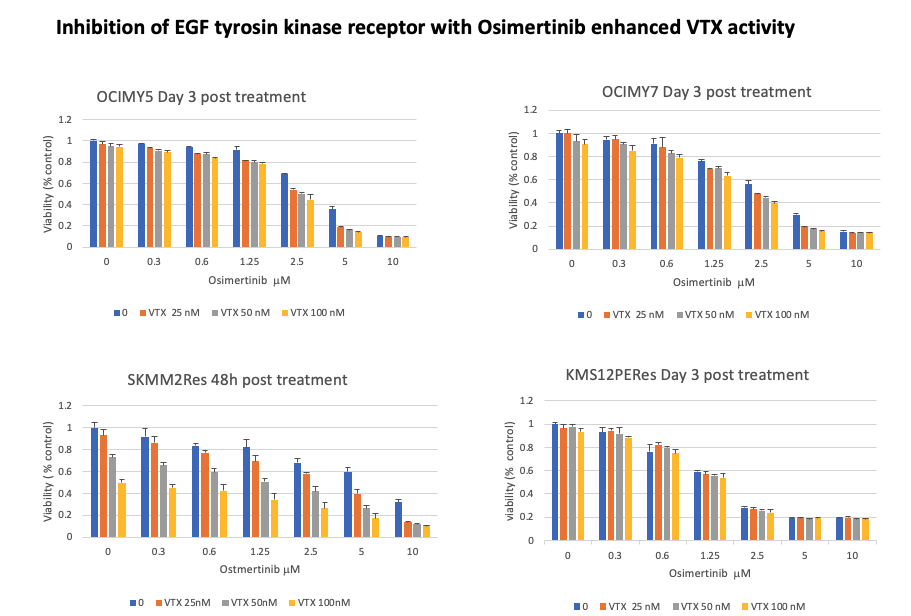

Supplement: Supplementary file 1 — Supplemental Figures and Tables [file 41408_2025_1215_MOESM1_ESM.docx]
